# COVID-19 pneumothorax in the UK: a prospective observational study using the ISARIC WHO clinical characterisation protocol

**DOI:** 10.1183/13993003.00929-2021

**Published:** 2021-09-16

**Authors:** Stefan J. Marciniak, James Farrell, Anthony Rostron, Ian Smith, Peter J.M. Openshaw, J. Kenneth Baillie, Annemarie Docherty, Malcolm G. Semple

**Affiliations:** 1 CIMR, University of Cambridge, Cambridge, UK; 2 Addenbrooke's Hospital, Cambridge, UK; 3 Royal Papworth Hospital, Cambridge, UK; 4 Usher Institute of Population Health Sciences and Informatics, University of Edinburgh, Edinburgh, UK; 5 Integrated Critical Care Unit, Sunderland Royal Hospital, South Tyneside and Sunderland NHS Foundation Trust, Sunderland, UK; 6 Translational and Clinical Research Institute, Newcastle University, Newcastle upon Tyne, UK; 7 South Tyneside and Sunderland NHS Foundation Trust, Sunderland, UK; 8 Respiratory Medicine, Imperial College London, London, UK; 9 Roslin Institute, University of Edinburgh, Edinburgh, UK; 10 MRC Human Genetics Unit, University of Edinburgh, Edinburgh, UK; 11 Intensive Care Unit, Royal Infirmary of Edinburgh, Edinburgh, UK; 12 NIHR Health Protection Research Unit in Emerging and Zoonotic Infection Institute of Infection and Global Health, University of Liverpool, Liverpool, UK; 13 Joint first authors

## Abstract

Pneumothorax is an important complication of coronavirus disease 2019 (COVID-19) [1, 2]. Based on a series of 60 individuals, we previously estimated that 0.91% of people admitted to hospital with COVID-19 develop pneumothorax [1]. Males accounted for three quarters of those affected, and patients requiring noninvasive or invasive ventilatory support appeared at elevated risk. In a separate series of ventilated patients with COVID-19, barotrauma, defined as pneumothorax or pneumomediastinum, was found to be an independent risk for death [2]. During the pandemic, treatment strategies have evolved, influenced by large randomised controlled trials and clinical experience. Following the landmark results from the RECOVERY trial [3], dexamethasone became standard of care for patients requiring supplemental oxygen. Following the first UK wave between March and June 2020, use of noninvasive respiratory support became more common [4, 5]. Such changes could plausibly alter the incidence of pneumothorax caused by COVID-19. Indeed, a recent small study reported an increase in pneumothoraces in the second wave of COVID-19 in Italy, leading to speculation that dexamethasone use might have been causal [6].

## Abstract 2

**Population level data from 131 679 patients show that COVID-19 pneumothorax occurs in 0.97% of admitted patients, especially males and smokers, and is associated with increased mortality** https://bit.ly/3oB27ez

*To the Editor:*

Pneumothorax is an important complication of coronavirus disease 2019 (COVID-19) [1, 2]. Based on a series of 60 individuals, we previously estimated that 0.91% of people admitted to hospital with COVID-19 develop pneumothorax [1]. Males accounted for three quarters of those affected, and patients requiring noninvasive or invasive ventilatory support appeared at elevated risk. In a separate series of ventilated patients with COVID-19, barotrauma, defined as pneumothorax or pneumomediastinum, was found to be an independent risk for death [2]. During the pandemic, treatment strategies have evolved, influenced by large randomised controlled trials and clinical experience. Following the landmark results from the RECOVERY trial [3], dexamethasone became standard of care for patients requiring supplemental oxygen. Following the first UK wave between March and June 2020, use of noninvasive respiratory support became more common [4, 5]. Such changes could plausibly alter the incidence of pneumothorax caused by COVID-19. Indeed, a recent small study reported an increase in pneumothoraces in the second wave of COVID-19 in Italy, leading to speculation that dexamethasone use might have been causal [6].

To examine COVID-19 pneumothorax at a population level during the first and second waves in the UK, we analysed data from the International Severe Acute Respiratory and emerging Infections Consortium (ISARIC) World Health Organization (WHO) Clinical Characterisation Protocol UK (CCP-UK). The study is being performed by the ISARIC Coronavirus Clinical Characterisation Consortium (ISARIC4C) in 311 hospitals across England, Scotland and Wales (National Institute for Health Research Clinical Research Network Central Portfolio Management System ID 14152) [7]. Approval was granted by the following ethics committees: South Central Oxford (Ref 13/SC/0149), Scotland (Ref 20/SS/0028), and WHO (RPC571, RPC572). Routine health data collation did not require consent.

From its activation on 17 January, 2020 to 15 February, 2021, 131 679 patients aged ≥18 years were recruited to CCP-UK if they were admitted to hospital with a positive SARS-CoV-2 (severe acute respiratory syndrome coronavirus 2) PCR or were considered highly clinically likely to have COVID-19. Of these, 1283 (0.97%) had a pneumothorax at some stage during their admission; 68.5% (879/1283) of those with a pneumothorax were male (figure 1a). Taking 15 August as the boundary between the first and second waves of the pandemic, 56.1% (720) of pneumothoraces occurred during the first wave with an overall incidence of 1.01% (720/70 969). During the first 6 months of the second wave, the incidence of pneumothorax was not significantly different at 0.93% (563/60 710; p=0.12).

**FIGURE 1:**
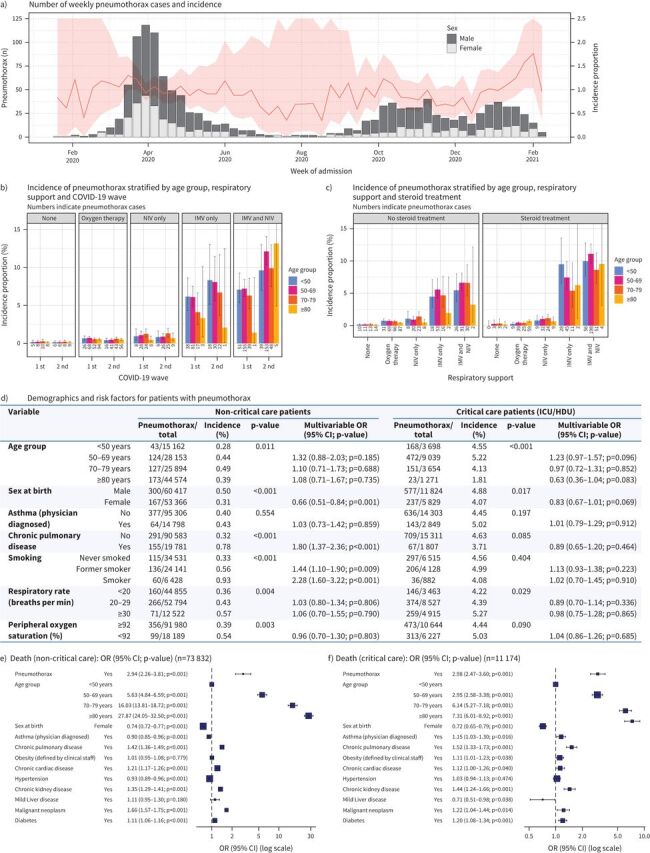
Coronavirus disease 2019 (COVID-19) pneumothorax in the ISARIC4C dataset. a) Histogram of incidence of pneumothoraces over time (absolute numbers, light grey females, dark grey males); line graph of pneumothorax incidence (percentage with 95% confidence interval). b) Incidence of pneumothorax by age, ventilatory support and pandemic wave. c) Incidence of pneumothorax by age, ventilatory support and steroid therapy: NIV: noninvasive respiratory support; IMV: invasive respiratory support. d) Demographics and risk factors for pneumothorax in non-critical care and critical care patients. Critical care patients include those admitted to an intensive care unit (ICU) or high dependency unit (HDU). Sample size for multivariable analysis was n=61 046 and 10 719 for non-critical care and critical care populations, respectively. e) Risk factors for death in non-critical care patients. f) Risk factors for death in critical care patients.

The incidence of pneumothorax differed between groups defined by the level of respiratory support they received. In patients requiring no supplemental oxygen, only 0.16% (60/37 030) had a pneumothorax; of those requiring oxygen without pressure support 0.56% (396/70 609) had pneumothoraces; treatment with noninvasive respiratory support alone was associated with an incidence of 0.96% (137/14 251); however, significantly more patients (6.1%; 195/3182) who received invasive ventilation also had a pneumothorax (p=0.004) (figure 1b). Patients who received both noninvasive respiratory support and invasive ventilation had the highest incidence of pneumothorax at 8.5% (491/5749; p<0.0001). It is noteworthy that, despite an overall similar incidence of pneumothoraces during the first and second waves, the incidence of pneumothorax in patients who received both noninvasive respiratory support and invasive ventilation during their admission was lower in the first than the second wave (6.9% *versus* 11.2%, p<0.001) (figure 1b). Among patients who did not receive corticosteroid treatment, 0.70% (598/85 961) developed a pneumothorax *versus* 1.46% (609/41 798) in those who did (p<0.001). Without steroids, pneumothoraces occurred in 4.94% (89/1800) of patients who received invasive ventilation *versus* 6.31% (149/2363) of patients who received both noninvasive respiratory support and invasive ventilation. Of patients treated with steroids, 7.57% (86/1136) of those treated with invasive ventilation *versus* 10.36% (309/2983) of those who received noninvasive respiratory support and invasive ventilation suffered pneumothoraces (figure 1c). However, after adjusting for respiratory severity on admission (respiratory rate and peripheral oxygen saturation) and comorbidities, corticosteroid therapy was not independently associated with increased incidence of pneumothorax either in critical care (intensive care unit (ICU) or high dependency unit) or non-critical care populations (p=0.20 and 0.65 respectively).

In patients on non-critical care wards, asthma was not associated with pneumothorax after adjustment for age, sex and respiratory severity on admission, although “chronic pulmonary disease” (which includes COPD, interstitial lung disease and sarcoidosis) was associated with pneumothorax with an odds ratio of 1.80 (95% CI 1.37–2.36; p<0.001) (figure 1d). Former and current smokers were at increased risk: OR 1.44, 95% CI 1.10–1.90 (p=0.009) and OR 2.28, 995% CI 1.60–3.22 (p<0.001), respectively (figure 1d). These associations did not translate to the critical care population, where respiratory comorbidities and smoking were not significant predictors of pneumothorax (figure 1d). Restricting the analysis to patients not on pressure support, respiratory severity was not associated with increased risk of pneumothorax in either non-critical care and critical care populations.

In this series, pneumothorax with COVID-19 was associated with a worse prognosis. Adjusted for age, sex and comorbidities, the odds ratio for death was 2.94 (95% CI 2.26–3.81; p<0.001) in non-critical care patients with pneumothorax compared to those without (figure 1e). For critical care patients, the adjusted odds ratio for death was 2.98 (95% CI 2.47–3.60; p<0.001) (figure 1f). Further analysis in both non-critical care and critical care populations showed that the odds ratio of interaction terms between pneumothorax, age, sex and comorbidities were not significant in predicting death. This suggests that the increased risk of death from pneumothorax was similar across subgroups.

There are several limitations to this study. The data collected from three London sites are excluded after 15 November, 2020 due to unreliable classification of ICU patients. Inevitably, there are missing data, but the relatively low numbers are unlikely to impact the results. The case report form (CRF) of ISARIC does not request information on the timing of pneumothoraces and so we are unable to establish whether pneumothoraces occurred following the introduction of ventilatory support or if the presence of a pneumothorax resulted in respiratory deterioration requiring intubation. The CRF does not include history of previous pneumothorax nor treatment of pneumothorax. The CRF does not include pneumomediastinum, so we are unable to comment on this related complication. The lack of randomisation in any observational study makes attribution of causality impossible. Therefore, we are unable to determine whether the increased incidence of pneumothorax in ventilated patients reflects severity of disease or is iatrogenic. In this regard, the RECOVERY-RS (Respiratory Support) trial may shed light on the relationship between ventilatory support and pneumothorax [8]. The place of noninvasive respiratory support requires further study, since those who failed to respond to this and then required invasive ventilation appeared at greater risk of pneumothorax than those who underwent early intubation and mechanical ventilation.

Pneumothorax and pneumomediastinum have emerged as important complications of COVID-19 [1, 2, 9]. Our previous smaller series suggested an incidence of 0.91% [1], similar to the 1.7% reported for severe acute respiratory syndrome (SARS) caused by SARS-CoV-1 [10]. We now confirm, using ISARIC data, which covers 40–45% of patients hospitalised for COVID-19 in England, Wales and Scotland, that 0.97% suffer a pneumothorax, but there are marked differences between subgroups. These may reflect disease severity, but we are unable to exclude an iatrogenic component. We report a clear association between incident pneumothorax and invasive mechanical ventilation. Pneumothorax had also been reported in patients with Middle East respiratory syndrome-related coronavirus infection, and as with SARS, pneumothorax was a marker of poor prognosis [10, 11]. Although in our previous small study we observed increased mortality only in patients >70 years of age, our current large series reveals that pneumothorax is independently associated with mortality over a wider age range.

In summary, we report data from the ISARIC4C study of 131 679 patients admitted with COVID-19 that reveal an overall incidence of pneumothorax of 0.97%. Male sex, smoking, chronic pulmonary disease and invasive ventilation were associated with increased risk of pneumothorax. Pneumothorax is associated with increased mortality in COVID-19.

## Shareable PDF

10.1183/13993003.00929-2021.Shareable1 This one-page PDF can be shared freely online.
Shareable PDF ERJ-00929-2021.Shareable
