# Supplementary material for: COVID-19 pneumothorax in the UK: a prospective observational study using the ISARIC WHO clinical characterisation protocol
Source: Eur Respir J. 2021 Sep 16;58(3):2100929. doi: 10.1183/13993003.00929-2021 (PMC8186005; doi:10.1183/13993003.00929-2021)
Supplement: Supplementary file 1 [file ERJ-00929-2021.Shareable.pdf]

EUROPEAN RESPIRATORY JOURNAL
RESEARCH LETTER
S.J. MARCINIAK ET AL.

SHAREABLE PDF

COVID-19 pneumothorax in the UK: a prospective observational study using the ISARIC WHO clinical characterisation protocol

Stefan J. Marciniak [1,2,3,13], James Farrell[4,13], Anthony Rostron [5,6,7], Ian Smith[3], Peter J.M. Openshaw [8], J. Kenneth Baillie [9,10,11], Annemarie Docherty[4] and Malcolm G. Semple [12]

[1]CIMR, University of Cambridge, Cambridge, UK. [2]Addenbrooke's Hospital, Cambridge, UK. [3]Royal Papworth Hospital, Cambridge, UK. [4]Usher Institute of Population Health Sciences and Informatics, University of Edinburgh, Edinburgh, UK. [5]Integrated Critical Care Unit, Sunderland Royal Hospital, South Tyneside and Sunderland NHS Foundation Trust, Sunderland, UK. [6]Translational and Clinical Research Institute, Newcastle University, Newcastle upon Tyne, UK. [7]South Tyneside and Sunderland NHS Foundation Trust, Sunderland, UK. [8]Respiratory Medicine, Imperial College London, London, UK. [9]Roslin Institute, University of Edinburgh, Edinburgh, UK. [10]MRC Human Genetics Unit, University of Edinburgh, Edinburgh, UK. [11]Intensive Care Unit, Royal Infirmary of Edinburgh, Edinburgh, UK. [12]NIHR Health Protection Research Unit in Emerging and Zoonotic Infection Institute of Infection and Global Health, University of Liverpool, Liverpool, UK. [13]Joint first authors.

Stefan J. Marciniak (sjm20@cam.ac.uk)

Shareable abstract (@ERSpublications)
Population level data from 131 679 patients show that COVID-19 pneumothorax occurs in 0.97% of admitted patients, especially males and smokers, and is associated with increased mortality https://bit.ly/3oB27ez

Cite this article as: Marciniak SJ, Farrell J, Rostron A, *et al*. COVID-19 pneumothorax in the UK: a prospective observational study using the ISARIC WHO clinical characterisation protocol. *Eur Respir J* 2021; 58: 2100929 [DOI: 10.1183/13993003.00929-2021].

This single-page version can be shared freely online.

Copyright ©The authors 2021.

This version is distributed under the terms of the Creative Commons Attribution Non-Commercial Licence 4.0. For commercial reproduction rights and permissions contact permissions@ersnet.org

Received: 29 March 2021
Accepted: 11 May 2021

To the Editor:

Pneumothorax is an important complication of coronavirus disease 2019 (COVID-19) [1, 2]. Based on a series of 60 individuals, we previously estimated that 0.91% of people admitted to hospital with COVID-19 develop pneumothorax [1]. Males accounted for three quarters of those affected, and patients requiring noninvasive or invasive ventilatory support appeared at elevated risk. In a separate series of ventilated patients with COVID-19, barotrauma, defined as pneumothorax or pneumomediastinum, was found to be an independent risk for death [2]. During the pandemic, treatment strategies have evolved, influenced by large randomised controlled trials and clinical experience. Following the landmark results from the RECOVERY trial [3], dexamethasone became standard of care for patients requiring supplemental oxygen. Following the first UK wave between March and June 2020, use of noninvasive respiratory support became more common [4, 5]. Such changes could plausibly alter the incidence of pneumothorax caused by COVID-19. Indeed, a recent small study reported an increase in pneumothoraces in the second wave of COVID-19 in Italy, leading to speculation that dexamethasone use might have been causal [6].
